# The distributional properties of exemplars affect category learning and generalization

**DOI:** 10.1038/s41598-021-90743-0

**Published:** 2021-05-28

**Authors:** Paulo F. Carvalho, Chi-hsin Chen, Chen Yu

**Affiliations:** 1 Human-Computer Interaction Institute, Carnegie Mellon University, Pittsburgh, PA USA; 2 Department of Otolaryngology-Head and Neck Surgery, The Ohio State University, Columbus, OH USA; 3 Department of Psychology, The University of Texas At Austin, Austin, TX USA

**Keywords:** Psychology, Human behaviour

## Abstract

What we learn about the world is affected by the input we receive. Many extant category learning studies use uniform distributions as input in which each exemplar in a category is presented the same number of times. Another common assumption on input used in previous studies is that exemplars from the same category form a roughly normal distribution. However, recent corpus studies suggest that real-world category input tends to be organized around skewed distributions. We conducted three experiments to examine the distributional properties of the input on category learning and generalization. Across all studies, skewed input distributions resulted in broader generalization than normal input distributions. Uniform distributions also resulted in broader generalization than normal input distributions. Our results not only suggest that current category learning theories may underestimate category generalization but also challenge current theories to explain category learning in the real world with skewed, instead of the normal or uniform distributions often used in experimental studies.

## Introduction

Category learning is critical to our day-to-day lives. Categories allow us to be able to act in the world by knowing what to expect from novel similar situations^1^. Many categories are learned by processing perceptual information perceived by our sensory systems. With sensory input, such as 2D object images projected on the retina or sound waves perceived by the ears, our cognitive system is able to sort and group a collection of visual object instances or sounds as belonging to the same category. There is a large literature on the perceptual and cognitive processes that support human category learning, e.g.^2,3^. Most studies focus on examining the ability to acquire novel categories through laboratory studies^1^. Although there is wide agreement that the sensory input the learning system receives influences the output of learning^4,5^, the role of the properties of the input distribution is less well-understood.

Corpus analyses suggest that the input from everyday environments is organized around distributions in which a few items have a much higher likelihood of being experienced, with a long tail of items that are experienced much less frequently^5,6,9–12^. For example, a child learns about a category representation of *dog* based on many exposures to her own dog encountered in everyday contexts along with fewer exposures of many other dogs encountered on other occasions. Such distributions are also true when looking at repetitions of specific items across time in infants’ early daily experience, e.g.^13^.

Given that input distributions in everyday environments seem to have one item that is seen more frequently among many category items, do the properties of the most frequent item relative to the other items in the sample matter? For instance, do learners represent the category differently when the most frequent item is highly similar to all the other items compared to input distributions in which the most frequent item is an extreme example of the category? Although previous research has made considerable progress in identifying how the learning input is created by the learner’s active information selection actions and organized around a highly frequent item^6,10,13,14^, it is still unknown if the properties of the distribution of exemplar items with different frequencies influence category representation and future generalization.

Category representation is intimately related to how learners generalize the category; according to most models of categorization, category decisions are made based on comparing the current item with the representation of previously categorized items. In exemplar models of categorization such as GCM^15^, ALCOVE^16^, or SUSTAIN^17^, the categorization of novel items is done by comparing the similarities of the novel item to those of stored items. The properties of the most frequent item in the input could have an influence on which properties of the stored items are used in the similarity comparisons. Thus, where the most frequent item is in the distributions could result in different category representations and, because of that, different generalizations.

We propose three hypotheses regarding how the properties of the most frequent item relative to the other items might influence later generalization. One possibility, which we will refer to as the Bias hypothesis, is that which item is most frequent has no impact on generalization. Because the cognitive system has a bias to represent input as normally distributed^18–20^, the output of the system will be a normally distributed generalization centered in the input space. That is, generalization will always match the bias of the system^20^.

Another possibility, which we will refer to as the Fidelity hypothesis, is that learners will represent the space differently with different input distributions in a way that accurately matches their input. That is, generalization will replicate the properties of the input and match it closely. Thus, the properties of the most frequent item in the input distribution completely determine the output distribution.

Finally, a third possibility, which we will refer to as the Mix hypothesis, is that the representation created by learners is the result of an interaction between the input provided and the cognitive system’s bias. In other words, the properties of the most frequent item of the input will have an effect on generalization by affecting how the systems’ bias is applied, e.g.^12,18,21–23^.

**Figure 1:**
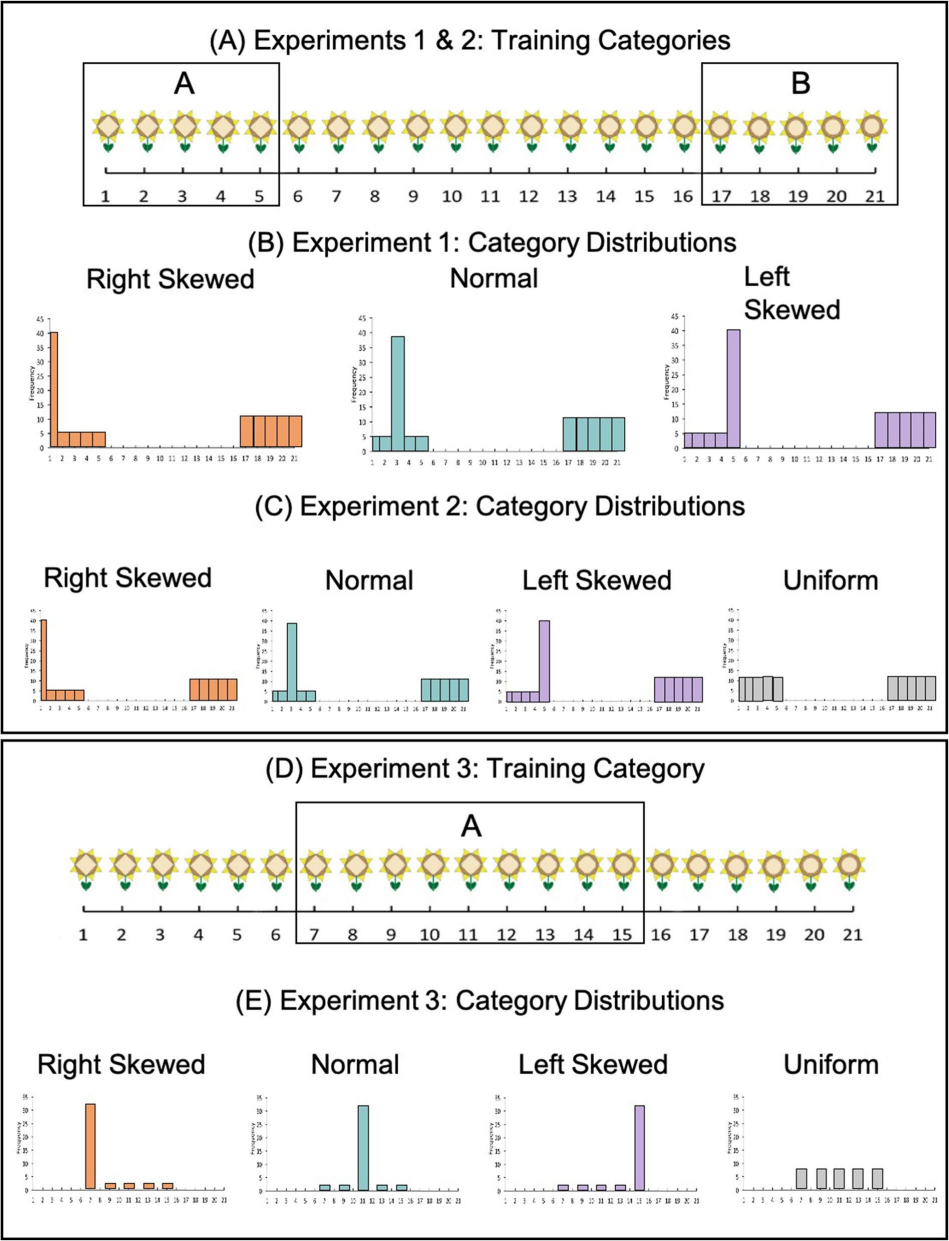
Schematic representations of the stimulus space used in Experiments 1 and 2 (**A**) and in Experiment 3 (**D**). Schematic representation of the input distributions in Experiment 1 (**B**), Experiment 2 (**C**), and Experiment 3 (**E**).

We will test these hypotheses by presenting learners with different input distributions in which the most frequent item is either central to the category (Normal Input; see Fig. 1B), thus maximally similar to all studied items, or peripheral (Skewed Input; see Fig. 1B), thus an extreme exemplar of the category. After training with different distributions, we will test participants' representations by asking them to categorize all items (both old and new) in a continuum and evaluate to what extent they generalize the category learned to novel items. Such a procedure allows us to evaluate—at the group level—the generalizations created by participants following each type of input.

Assuming that each studied item has some level of activation related to its evidentiary strength towards a given category in the system, and that this activation will influence how much categorization is generalized to novel items, e.g.^16–18^, the three hypotheses highlighted make clear distinct predictions.

Conversely, if the Fidelity hypothesis is correct, activation will match the input resulting in maximum activation for the most frequent item. Thus, generalization will be wider when the peak of the distribution is closest to the category boundary. Finally, if the Mix hypothesis is correct, activation will be normally distributed (the bias of the system) but shifted towards the most frequent item in the input (which might not be the center of the input) and encompass all studied items. This will result in an activation gradient that is wider and shifted towards the most frequent item (peak of the distribution) in the case of a skewed input distribution (see Fig. 1B). Therefore, the generalization will be wider for the Skewed input.

### The current studies

In the first study, we taught participants two categories of novel objects, one using one of three unimodal input distributions: right-skewed, normal, or left-skewed distributions, and another using a uniform input distribution. We used a uniform distribution for the second category to keep constant the properties of the second category and guarantee that any difference found was due to the characteristics of the unimodal distribution only. Following category learning, we compared learners’ generalization after experiencing different unimodal distributions. To this end, we determined the extent of categorization (broadness) by comparing the categorization performance for each type of distribution.

In two follow-up studies we investigated alternative explanations for the results found in Experiment 1. In Experiment 2, we tested whether other properties of the distributions (the steepness of the distributions) affected learners’ generalization patterns and the impact of the tail of the distribution by including a uniform target distribution. Finally, in Experiment 3 we directly compared generalization from training data generated from a single distribution, either skewed, normal, or uniform, that did not require the inclusion of a second category.

## Experiment 1

### Method

#### Participants

We used G*Power^24^ to calculate the required sample size to detect a small effect size (*f* = 0.15) for the within-subject effect of type of input distribution, considering 2 between-subject groups (given counterbalancing conditions, see details below) and 3 levels of within-subject measurement, with $\alpha = 0.05$. The sample size required to achieve 75% power was 66 participants (assuming a correlation among repeated measures of 0.5 and a nonsphericity correction of 1). Because of the limited previous research to provide a precise estimate of expected effect size, we decided to collect data from more than the minimum number of participants described above, approximately 80 participants to accommodate for potential dropout. We did not analyze the data before completing data collection with the target sample size.

84 volunteer undergraduate students at Indiana University (48 females, mean age: 19) participated in this study in return for course credit. However, due to technical problems, 3 participants did not finish the experiment and were excluded from the analyses. 81 participants completed all conditions. Data from one participant was excluded from analyses due to not following instructions (responding with the same key to all test trials). Recruitment and experimental procedures were approved by Indiana University’s Institutional Review Board and all experiments were performed in accordance with relevant guidelines and regulations. All participants gave informed consent prior to participation.

#### Design and procedure

The stimuli were sets of novel-looking flowers, each containing 21 flowers; there were 3 different sets, all with the same properties but different visual appearance. Objects in each set varied along a continuum in one perceptual feature (shape of the disk, the color of petals, the length of the stem). Take Fig. 1A as an example, the flower on the left extreme has a diamond-shaped disk whereas the flower on the right extreme has a circle-shaped disk. The training items were the 5 most extreme items on each side (e.g., items 1–5 with a more diamond-like shape and items 17–21 with a more circle-like shape).

There were two training categories, one with a uniform distribution and the other with a unimodal distribution (Fig. 1B). There were 3 unimodal distribution conditions, a right-skewed distribution with a peak at the exterior of the continuum, a normal distribution with a peak in the middle, and a left-skewed distribution with a peak in the interior of the continuum. We included two skewed distributions because, although our prediction pertains only to the distinction between whether the most frequent item is maximally similar to all studied items (normal distribution) or an extreme exemplar (skewed distribution), it is possible that the similarity of that item to a different category has an impact as well. For example, it is possible that seeing more frequently an item that is maximally dissimilar to the other category (right skewed distribution), creates a strong anchoring effect thus increasing category broadness, e.g.^25,26^. Similarly, it is also possible that when the most frequent item is more similar to the other category it broadens the representation of the target category because it is more similar to items in the other category.

For each set, which side (items 1–5 or 17–21) was the uniform or unimodal distribution was counterbalanced across participants. The members in each category occurred 60 times in total. Members in the uniform distribution each occurred 12 times. One member in each of the skewed distributions occurred 40 times while the other members occurred 5 times each.

In each training trial, participants saw one of the training flowers on the computer screen and had to guess whether it belonged to category A or B. After they selected one of the categories, they saw a feedback sentence “The correct answer is ___.” After going through the training phase, participants were tested in 21 trials, each containing one of the flowers in the continuum. In each test trial, participants were asked to select which category the flower belonged to. However, unlike the training phase, no feedback was provided in the test phase.

### Results and discussion

**Figure 2:**
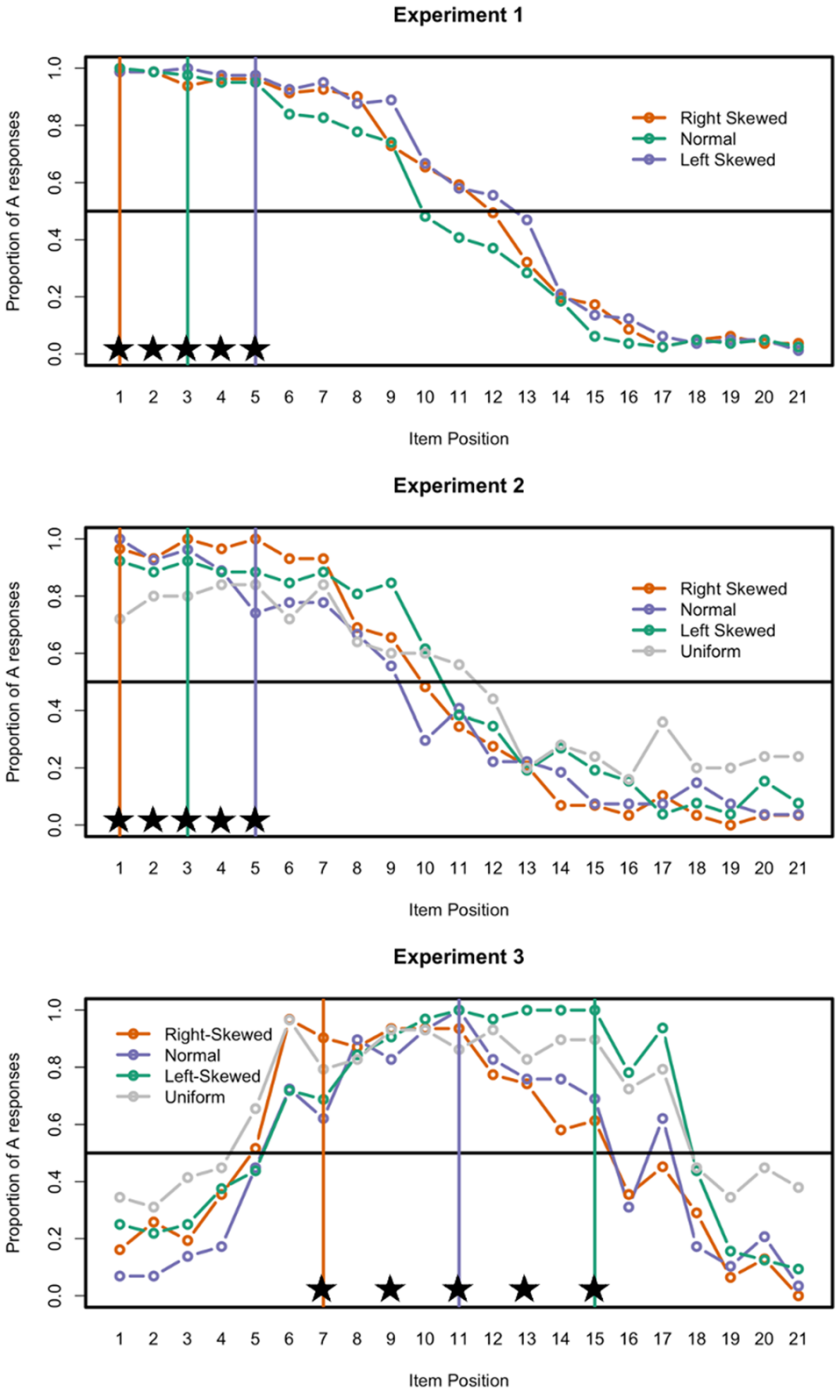
Proportion of “A” responses during the test for each experiment (each row from top to bottom: Experiment 1, Experiment 2, and Experiment 3) and study distribution conditions. Vertical lines indicate the peak item for the study distribution in each condition, the horizontal line indicates 50% A responses, and the stars indicate studied items.

The main question of interest is whether category representation is affected by the category distribution. Specifically, we asked whether a skewed input distribution, as opposed to a normal distribution of items, during study influenced how broadly participants extended the learned category to new items. To do so, we started by transforming the participants’ responses during the test phase so that the category learned with a uniform distribution was always on the right and the one with the unimodal distribution was on the left, and such that responses on the left side corresponded to category A and on the right to category B (Fig. 2). Therefore, the right-skewed distribution had the peak on the most extreme left side of the continuum whereas the left-skewed distribution had the peak closer to the center of the continuum (and thus closer to the other training category). This step was important so that participants and conditions could be directly compared.

The plot on the top panel of Fig. 2 shows the proportion of “A” responses in each condition during the test phase. As it can be seen in the figure, the type of distribution changes how the categories are represented. Specifically, participants who saw a skewed distribution of the category items during training had a wider representation of the category compared to those who trained with normally distributed sample of items.

To quantify the observed impact of different distributions on participants’ representations of the categories, we estimated the furthest item in the continuum participants were still likely to accept as a member of category A, i.e., the category change-point. This is a measure of how broad the category is. To estimate the change-point, we identified, for each participant, the highest value classified as A (e.g., flower 14) and the lowest value classified as B (e.g., flower 10). The mid-point between these two values (in the example, (14–10)/2 + 10, so flower 12) approximates the boundary between the two categories for that participant. Note that it is possible that a participant did not consistently classify the objects between the two values as A or B, that is, some items might be classified as A and others as B, which is consistent with the idea that this is the transition area between the two categories, e.g.^27^. In the statistical analyses below, we used a one-way ANOVA with input distribution (left-skewed, right-skewed, normal) as a within-subject factor predicting changing point. All analyses were followed-up with two planned contrasts to (1) test the Bias hypothesis by comparing generalization following training with skewed distributions and generalization following normally distributed input. If the Bias hypothesis is correct, we should see no difference between these two types of input distributions. In addition, (2) we test the Fidelity hypothesis by comparing generalization following training with Right-Skewed distributions (Peak at 1) with Normal distributions (Peak at 3). If the Fidelity hypothesis is correct, then the Normal distribution should result in broader generalization than the Right-Skewed distribution.

How far participants generalized the categories varied across conditions (see Table 1, see also Supplementary Information for plots), $F(\mathrm{2,158})=6.88, p=.001,{\eta }_{G}^{2}=0.001$. Planned contrasts comparing the effect of skewed vs. normal distributions showed that participants extended their category further when a skewed distribution was used during study,$t (158) =3.43, p=.002$. Participants also created slightly broader categories after experiencing a Right-Skewed distribution of items during study compared to a Normal distribution, although this effect was only marginally statistically significant, $t (158) =-1.88, p=.062$. This pattern suggests that generalization following Normal input is the same or slightly narrower than following Right-Skewed input. Overall, the results of this study suggest that the distribution of items during category learning influences the category representation that learners exhibit. In other words, the representation distribution that participants created does not match the input distribution they received. Overall, the output distribution evident from the generalization analyses seems to match the predictions of the Mix Hypothesis—it is not consistent with either the Fidelity or the Bias hypotheses.

**Table 1:** Mean change-point (95% confidence intervals) for each condition in Experiment 1.

	Measure	Right-skewed	Normal	Left-skewed	*F*	*p-value*
Exp. 1	Change-point	11.26 (10.61–11.91)	10.58 (9.98–11.19)	11.96 (11.51–12.41)	$6.88$	.001

## Experiment 2

The main goal of Experiment 2 was to replicate and extend the results of Experiment 1 to skewed distributions with different properties. Specifically, we manipulated the steepness of the skewed distributions by changing the ratio between the most frequent item and the “tail” items. In Experiment 1, we used distributions with a 40:5 steepness ratio, that is, the most frequent item was presented 40 times whereas the remaining four items were presented 5 times each. Thus, the more frequent item is 8 times more frequent than the remaining items. It is possible that such a mild steepness ratio influenced the results, and a different pattern would be found if we used more extreme steepness ratios. For example, it is possible that with larger steepness ratios the difference between skewed and normally distributed input would not be present, which would lead to the conclusion that the effect observed in Experiment 1 is tied to mild steepness, i.e., flatter distributions. Conversely, if the effect is due to skewness itself, then even if we increase the ratio, we should see the same effects. Therefore, in Experiment 2, we compared two different steepness ratios: 28:3 and 32:2, an almost 100% increase in steepness (from 9.3 to 16).

Although the two steepness ratios are equated for the total number of exposures to the category (40 presentations of each category), this number differs from that of Experiment 1 (60 presentations of each category). There were two reasons for this change: (1) it made the task shorter, which allowed us to expand the population from the undergraduate sample used in Experiment 1 to a broader online population, and (2) it allowed us to guarantee that the effect found in Experiment 1 is not tied to the total number of exposures.

Experiment 2 maintains the main manipulation of Experiment 1: using each of the steepness conditions we created three distribution conditions: right-skewed, normal, and left-skewed distributions, by manipulating whether the peak was on the left, center, or right of the sample (as in Experiment 1). Additionally, this manipulation was introduced as a between-subject design to reduce the possibility of the mutual influence of each condition on the others.

Finally, to further test the impact that the “tail” of the input distribution has on generalization, we also added a condition where both categories were presented using a uniform distribution. This new condition allows us to directly compare the generalization of each type of unimodal distribution with the uniform distribution. Because uniform distributions have more items at the extremes than a normal distribution, the Mix hypothesis would predict that the generalization would be broader following training with uniform input than normal input. The higher frequency of the extreme items in the uniform distribution combined with the systems’ central distribution bias should result in a wider output distribution that encompasses all strong pieces of evidence in the uniform input distribution.

In sum, the changes introduced in Experiment 2 relative to Experiment 1 allow us to test the generalizability of the findings in Experiment 1 to different steepness ratios, the total number of category exposures, different populations, and different experimental setups (from within-subject design to a between-subject design), as well as to directly compare generalization between unimodal and uniform distributions.

### Method

#### Participants

Using G*Power we determined that the sample size required to detect an effect size of $${\eta }_{G}^{2}=0.05$$(*f* = 0.31) with three distributions as in Experiment 1 in a between-subject design with 75% power was approximately 75 participants. We exceeded this goal to account for potential dropout, initially recruiting 82 participants randomly assigned to either 28:3 or 32:2 steepness ratio and either right-skewed, left-skewed, or normal distribution conditions. Recruitment for the fourth distribution condition (uniform distribution) was conducted after data collection for the other conditions had concluded (at the suggestion of a reviewer). Following the initial power calculation, we recruited an additional 26 participants for that condition. In total, we recruited 108 adults via Amazon Mechanical Turk (50 females, mean age: 33.32) to participate in this study. The entire study took approximately 5 min and participants were paid $0.60 for their participation. Participants were randomly assigned to one of 7 conditions, crossing the distribution steepness ratio (28:3 and 32:2) and the shape of the input distribution (right-skewed, normal, left-skewed) with an additional condition in which the input distribution was uniform (and therefore, had no steepness ratio). Recruitment and experimental procedures were approved by Indiana University’s Institutional Review Board and all participants gave informed consent prior to participation.

#### Procedure

Two steepness ratio conditions were used, 28:3 and 32:2. In both conditions, the training procedure was the same as that of Experiment 1, except that each category was trained 40 times.

In the 28:3 condition, each item in the uniform condition occurred 8 times. The peak item in the unimodal condition occurred 28 times whereas the rest of the training items occurred 3 times each. There were 3 distribution conditions: right-skewed, normal, and left-skewed distribution.

Similarly, in the 32:2 condition, each item in the uniform condition occurred 8 times. The peak item in the unimodal condition occurred 32 times whereas the rest of the training items occurred 2 times each. Again, there were 3 distribution conditions: right-skewed, normal, and left-skewed distribution (see Fig. 1C).

For the uniform distribution condition, every item in both categories was studied 8 times (see Fig. 1C). Half of the participants in this condition were randomly assigned to have the target category on the left and the other half on the right. Note that there were no differences between the two categories and this distinction is for the purposes of comparing with the other conditions only.

### Results and discussion

We used the same general analytic approach as in Experiment 1. Our planned contrasts directly test the three hypotheses. To test whether the Bias hypothesis is correct, we compare the effect of skewness on category generalization by contrasting the change-point following training with skewed distributions (Left-skewed and Right-Skewed) and non-skewed distributions (Normal and Uniform Distributions). If the Bias hypothesis is correct, then there should be no difference between these two types of distributions (skewed vs. non-skewed) because every input distribution will result in the same output. To test whether the Fidelity hypothesis is correct, we compare the Right-Skewed distribution and the Normal distribution (see Fig. 1C). If the Fidelity hypothesis is correct, then the Normal distribution input should result in broader generalization than the Right-skewed distribution. Finally, to test whether the Mix hypothesis is correct, we compare generalization following training with Normal and Uniform distributions. If the Mix hypothesis is correct, then we should see broader generalization with the Uniform distribution because the frequency of the edges of the input distribution is higher. In other words, a mix of the cognitive system’s bias towards normal representation and input that includes more evidence on the edges would result in a wider distribution with Uniform input than Normal input.

First, we looked at the effect of the steepness of the distribution on how broadly participants generalized the categories using the change-point measure, using a two-way ANOVA with ratio (28:3 vs 32:2) and type of distribution (Right-skewed vs. Normal vs. Left-Skewed) as between-subject factors. Because the Uniform condition does not have a more frequent item it does not have a ratio and therefore was not included in these analyses. The effect of steepness was not statistically significant, $F(\mathrm{1,76})=2.32, p=.132,{\eta }_{G}^{2}=0.03$, or interacted with the other variables, *F* < 1. Because there was no effect of steepness, we analyzed both steepness conditions together in the remainder of the analyses.

The plot in the middle panel of Fig. 2 shows the proportion of “A” responses during the test phase (combining both steepness conditions), showing a pattern similar to Experiment 1 (top panel of Fig. 2). Analyses using one-way ANOVA with type of input distribution as a between-subject factor to predict the extent of generalization (see Table 2 and Supplementary Information for plots) also show a similar pattern to Experiment 1. Overall, the distribution of the category during study influenced the category representation, $F(\mathrm{3,103})= 4.45, p=.006,{\eta }_{G}^{2}=0.11$. To explore this effect, we conducted a series of planned contrasts. Participants extended their categorization further with skewed when compared to non-skewed distributions, $t (103) =2.21, p=.029$, further with the right-skewed when compared to the normal distribution, $t (103) =2.19, p=.031$ , and further with the uniform distribution than the normal distribution, $t (103) =3.54, p<.0001$.

**Table 2:** Mean change-point (95% confidence intervals) for each condition in Experiment 2.

Measure	Right-Skewed	Normal	Left-skewed	Uniform	*F*	*p*-value
Change-point	10.03 (9.28–10.79)	9.51 (8.70–10.34)	11.25 (10.34–12.16)	11.12 (7.31–14.93)	4.45	.006

In sum, the results of Experiment 2 replicate the results of Experiment 1. Furthermore, the results of Experiment 2 suggest that the effect of skewed input distributions on output created from learning is not critically connected to the ratio between most and least frequent items used in the skewed distributions.

## Experiment 3

The results of the previous two studies suggest that the distribution of items during category learning influences the category representation that learners acquire. However, it is possible that the existence of a contrasting second category with a uniform distribution influenced the results. Because the uniform distribution was used in one extreme of the space, it was not possible to determine whether the distribution of items during study influences generalization independently from the distribution of the other categories in the space. To extend the results of Experiments 1 and 2, in this third experiment we used a single category learning task and compared the category representations participants created from skewed vs. non-skewed distributions.

### Method

#### Participants

Using G*Power we determined that the sample size required to detect an effect size of $${\eta }_{G}^{2}=0.05$$(*d* = 0.62) as in Experiments 1 and 2 with 75% power was approximately 84 participants. Initial recruitment for the three conditions (left-skewed, normal, and right-skewed input distributions) included 92 participants. The fourth condition (uniform input distribution) was added after recruitment and data analyses was completed for the other conditions (at the suggestion of a reviewer). We recruited an additional 29 participants for that additional condition. 121 adults recruited via Amazon Mechanical Turk participated in this study (45 females, mean age: 36.30). The entire study took approximately 5 min and participants were paid $0.60 for their participation. Recruitment and experimental procedures were approved by Indiana University’s Institutional Review Board and all participants gave informed consent prior to participation.

#### Design and procedure

Like in Experiments 1 and 2, the stimuli used in Experiment 3 were 21 flowers. However, instead of having the most extreme items as the training objects, the training items in Experiment 3 were items in the middle of the continuum. In addition, in order to increase the perceptual differences among items, instead of using the 5 items in the middle, we spread out the distance between training items and used items 7, 9, 11, 13, and 15 (see Fig. 1D,E). Thus, unlike the two previous experiments, in Experiment 3 in addition to the training items (items 7, 9, 11, 13, and 15) some novel items were covered by the training category boundary (e.g., items 8, 10, 12, and 14) while other novel items were outside of the training boundary (i.e., items more extreme than items 7 or 15).

The members in the training category occurred 40 times in total with a 32:2 steepness ratio (as in Experiment 2). One member in the distribution occurred 32 times while the other members occurred 2 times each. There were 4 distribution conditions, right-skewed distribution (i.e., peak at item 7), normal distribution (i.e., peak at item 11), left-skewed distribution (i.e., peak at item 15), and uniform distribution (i.e., no peak item).

In each training trial, participants saw one of the training flowers and a label “A” under it. Participants could take as long as they wanted before they clicked a button “next,” which started the presentation of the next trial. Similar to Experiments 1 and 2, after going through the training phase, participants were tested in 21 trials. Participants saw one flower in each test trial and were asked to click whether the flower was an “A” or “Not A” flower. No feedback was provided in the test phase.

### Results and discussion

We followed the same analytical approach as in Experiment 2 with the following exceptions. Because participants could generalize the category towards both the left and the right side, we calculated two category change-points, one on the left side of the stimuli continuum and one on the right side. When participants classified all items on one side as “A,” we estimated the change-point as the maximum possible in the continuum (1 or 21, depending on the side). To determine how broad the categories that learners acquired were, we calculated the difference between the two change-points (change-point right—change-point left). The bottom panel of Fig. 2 shows the probability of “A” responses during test for Experiment 3. As can be seen from the figure, the type of distribution, had an impact on how broad the category representation is $F\left(\mathrm{3,117}\right)= 6.81, p=.0003,{\eta }_{G}^{2}=0.15$ (see Table 3 and Supplementary Information). To explore this effect, we conducted a series of planned contrasts. There is a trend suggesting that participants extended their categorization further with skewed when compared to non-skewed distributions, $t (117) =0.93, p=.084$. Moreover, participants’ generalization was broader after training with right-skewed compared to normal distributions,$t (117) =2.10, p=.017$. Participants also created broader generalizations following training with uniform distributions compared to normal distributions, $t (117) =2.77, p<.0001$.

**Table 3:** Mean broadness (95% confidence intervals) of distribution for each condition in Experiment 3.

Measure	Right-skewed	Normal	Left-skewed	Uniform	*F*	*p-*value
Broadness	10.44 (4.24–16.63)	10.00 (3.50–16.49)	12.53 (5.32–19.75)	13.44 (5.84–21.05)	6.81	.0003

Overall, the results of this experiment replicate the results of Experiments 1 and 2 and suggest that the influence of skewed input distributions on output distributions is not critically tied to the existence of a second distribution.

## General discussion

Corpus analyses of everyday experiences suggest that most experience with categories is unimodally distributed, with some exemplars experienced more frequently than others. However, the learning consequences of the properties of this more frequently experienced exemplar relative to the other exemplars of the category is not well-understood. Across three studies, we showed that learning with input distributions in which the most frequent item is an extreme exemplar of the category (Skewed Distribution) results in broader category representation compared to input distributions where the most frequent item is maximally similar to all studied exemplars (Normal Distribution). Moreover, we showed in Experiments 2 and 3, that uniform input distributions (where items at the edges of the input are equally frequent) result in broader generalization than normal input distributions (where items at the edges of the distributions are less frequent).

We proposed three hypotheses whereby the similarity properties of the most frequent item relative to the rest of the category members could affect later generalization: the Bias hypothesis, the Fidelity hypothesis, and the Mix hypothesis. According to the Bias hypothesis, the output distribution will match the bias of the cognitive system—previously described as a normally distributed representation centered around the middle of the input distribution^18–20^. Thus, according to this hypothesis, whether the input distribution is skewed or normally distributed should have no discernible effect on how learners represent the categories and, by hypothesis, no effect on generalization.

According to the Fidelity hypothesis, the output distribution will match the input distribution. Thus, according to this hypothesis, the characteristics of the input distribution will influence how learners represent the categories, resulting in broader categories for normally distributed than Right-skewed distributions.

Finally, according to the Mix hypothesis, the output distribution will be a combination of the system’s bias and the properties of the input distribution. Thus, according to this hypothesis, the input distribution will influence how learners represent the categories, resulting in broader categories for skewed and uniform distributions compared to normal distributions.

Overall, the empirical results across the three experiments presented here are consistent with the Mix hypothesis. We found that the input distribution did have an impact on learners’ generalization (falsifying the Bias Hypothesis), that right-skewed distributions resulted in broader generalization than normal distributions (falsifying the Fidelity Hypothesis), and that uniform distributions resulted in broader generalization than normal distributions (consistent with the Mix Hypothesis).

Overall, these results are consistent with previous proposals that learners’ mental representation of categories is biased towards the mean of the input they received^19^ but see^20,23^. However, our empirical results also suggest that learners’ generalization is not *solely* the result of an existing bias towards the mean of the studied category space. We find that right-skewed distributions—for which the mean is *further* away from the center of the stimuli space—resulted in broader categories as well.

Why would a mix of a preexisting bias and the characteristics of the input result in broader generalizations for skewed distributions? One possible explanation is that learners represent the category as a skewed distribution (matching the input) but centered around the mean of the input (matching the bias). This would result in a representation that encompasses all studied items, but with higher activation closer to the peak of the input and slower decay towards the opposite side of the peak. Accordingly, we found broader generalization for skewed distributions where the peak of the distribution is at one side of the space, particularly, when the peak was closest to the category boundary. This is because, by hypothesis, the tail distance from the peak of the distribution led to broadening of the normal representation bias. Moreover, we found that uniform input distributions also resulted in broader generalization than a normal distribution. This is because, by hypothesis, the evidence at the extremes of the distribution was stronger than in the normal distribution, broadening the normal representation bias.

Another possibility is related to how skewness might influence *perceived* variance of the input. It has been demonstrated that participants create broader categories when the studied items are perceptually more variable^18,22,28^. In the present work, we maintained the properties of the items constant in such a way that all conditions had the same training stimuli and thus did not directly manipulate input variance. However, it is plausible to assume that the distribution affects the perceived variance of the study exemplars, that is, how variable the stimulus space is perceived to be. When the distance between the most frequent item and the last item from the tail is larger (as is the case with skewed distributions), or there are more items at the extremes of the distribution (as is the case with uniform distributions), participants might perceive the category as more variable (despite that being controlled for). This *perceived* variance could contribute to broader generalization with skewed distributions without any manipulation of stimuli variance, see e.g.^18^.

We should also note that previous work in category learning has investigated the related effects of item frequency and item perceptual variability on category learning. For example, Nosofsky^29^ demonstrated that generalization and typicality judgments are biased towards more frequently seen items in the input, see also^30^. However, in this paper, we investigate not only the effect of frequency (the peak of the distribution), but also its position relative to the other studied items. If generalization were pulled towards the most frequent item and its relative position had no effect, then we should see narrower generalizations for the right-skewed distribution than all the other distributions. Instead, we found that both skewed distributions resulted in wider generalization than the normal distribution (for which the peak is equally frequent but located in the middle relative to other studied items). These results suggest that more than only the item frequency matters for category learning and generalization—the skewness of the distribution also plays a role.

One way to integrate the current results and the Mix hypothesis into current theories of learning is to investigate how existing models of category learning could be adapted to fit the current results. Although our focus is not to compare current category learning models and their predictions, we believe that exemplar models of category learning could account for these effects by incorporating the frequency and order of studied items into similarity calculations. For example, the Sequential Attention Theory Model (SAT-M)^32^, an exemplar model of categorization, weighs feature relevance for categorization based on the sequence of events and more frequent features are weighted more heavily because they are present in more comparisons.

Although everybody would agree that children’s initial visual and auditory input creates the basis for what they can learn^4^, the relevant data for learning are not the statistics of the physical and social world but only the samples that emerge from the learners’ exploration and experiences. That is, the sampling process is implemented through the learner’s actions, creating their own data with unique properties and distributions^6^. For example, decades of laboratory studies have focused on questions regarding whether more varied or more frequent but less varied instances are optimal for learning^7^. Yet, recent computational evidence suggests that a combination of both—created by children’s natural interactions with the world—is, in fact, optimal for learning^5,8^. Importantly, corpus analyses suggesting that everyday input is not normally distributed but instead follows a skewed distribution are based on self-generated distributions created by the learner^8,10^ . Importantly, it has been shown before that active exploration of the space changes the learning outcomes and the properties of the input^31^. In our experiments learners did not control the input or its distribution. Thus, although our work suggests a clear effect of the properties of the most frequent item, it remains an open question whether this effect would interact with active and self-generated input exploration.

Our findings confirm previous suggestions that the output of learning does not match the input provided with potential consequences for theory and practice. Our findings go one step further by showing that the relative perceptual properties of the most frequent and least frequent items have an impact on future generalization. In everyday experience, this is likely to have a large effect in how categories are formed and represented. For example, when children see a skewed input distribution of objects, where there is a clearly most frequent item (their favorite sippy cup) but a long tail of substantially less frequent items of the same category (wine glasses) they are creating broad, more inclusive categories. This early process might potentiate early category learning by establishing fewer categories that need to be learned. The same process might also be a potential cause for the well-established overgeneralization errors in young children, e.g.^33^. By creating broader categories from skewed distributions, children might include as part of the category more perceptually distant objects. Consequently, children will not only have fewer categories but also include more distant objects as part of existing categories—thus resulting in overgeneralization.

Finally, the work presented here highlights the importance of studying how information is distributed in the real world. Instead of working on theoretical assumptions about the input, we need to develop experiments, theories, and models that empirically reflect real-world distributions. Because different distributions in the input may engage different learning mechanisms or the same mechanism in different ways, and as a result, create different learning outcomes as demonstrated in the present study, the conclusions we arrive at based on real-world input might differ substantially from the current theories of category learning.

## Supplementary Information

Supplementary Information.
