# Supplementary material for: The distributional properties of exemplars affect category learning and generalization
Source: Sci Rep. 2021 May 28;11:11263. doi: 10.1038/s41598-021-90743-0 (PMC8163832; doi:10.1038/s41598-021-90743-0)
Supplement: Supplementary file 1 — Supplementary Information. [file 41598_2021_90743_MOESM1_ESM.docx]

**Supplementary Materials**

**
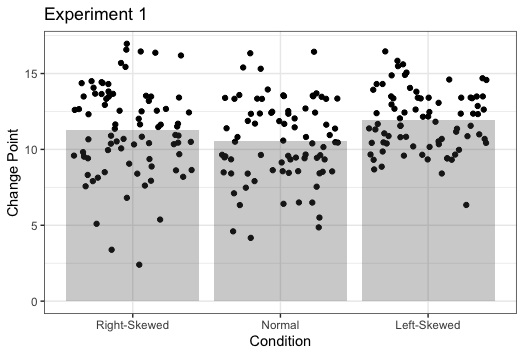
**

**Figure S1:** Mean change-point (bars) and individual participant change-points (dots) for each condition in Experiment 1. All participants completed all conditions.

**
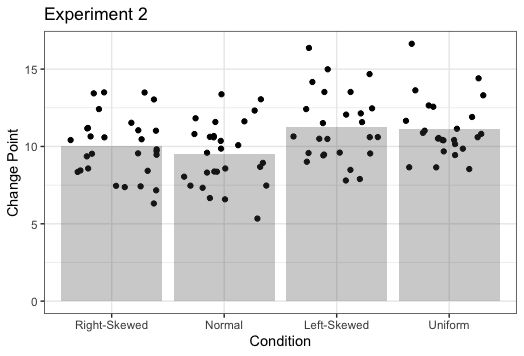
**

**Figure S2:** Mean change-point (bars) and individual participant change-points (dots) for each condition in Experiment 2. Participants were randomly assigned to only one condition.

**
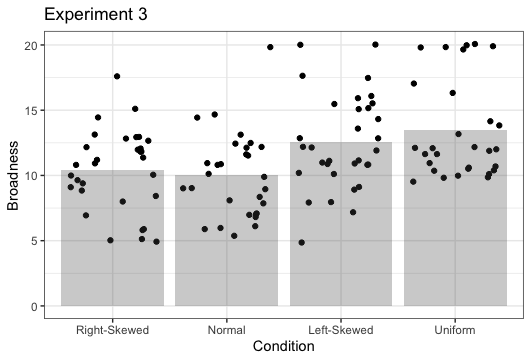
**

**Figure S3:** Mean change-point (bars) and individual participant change-points (dots) for each condition in Experiment 3. Participants were randomly assigned to only one condition.
